# Towards a Clinically Relevant Lentiviral Transduction Protocol for Primary Human CD34^+^ Hematopoietic Stem/Progenitor Cells

**DOI:** 10.1371/journal.pone.0006461

**Published:** 2009-07-30

**Authors:** Michelle Millington, Allison Arndt, Maureen Boyd, Tanya Applegate, Sylvie Shen

**Affiliations:** Johnson and Johnson Research Pty Ltd., Eveleigh, New South Wales, Australia; University of Michigan, United States of America

## Abstract

**Background:**

Hematopoietic stem cells (HSC), in particular mobilized peripheral blood stem cells, represent an attractive target for cell and gene therapy. Efficient gene delivery into these target cells without compromising self-renewal and multi-potency is crucial for the success of gene therapy. We investigated factors involved in the *ex vivo* transduction of CD34^+^ HSCs in order to develop a clinically relevant transduction protocol for gene delivery. Specifically sought was a protocol that allows for efficient transduction with minimal *ex vivo* manipulation without serum or other reagents of animal origin.

**Methodology/Principal Findings:**

Using commercially available G-CSF mobilized peripheral blood (PB) CD34^+^ cells as the most clinically relevant target, we systematically examined factors including the use of serum, cytokine combinations, pre-stimulation time, multiplicity of infection (MOI), transduction duration and the use of spinoculation and/or retronectin. A self-inactivating lentiviral vector (SIN-LV) carrying enhanced green fluorescent protein (GFP) was used as the gene delivery vehicle. HSCs were monitored for transduction efficiency, surface marker expression and cellular function. We were able to demonstrate that efficient gene transduction can be achieved with minimal *ex vivo* manipulation while maintaining the cellular function of transduced HSCs without serum or other reagents of animal origin.

**Conclusions/Significance:**

This study helps to better define factors relevant towards developing a standard clinical protocol for the delivery of SIN-LV into CD34^+^ cells.

## Introduction

Gene therapy holds promise for the cure of various inherited and acquired diseases, as evidenced by the success in the treatment of X-linked severe combined immunodeficiency (SCID-X1) [1], adenosine deaminase (ADA) deficiency [2] and chronic granulomatous disease (CGD) [3]. HSCs are potential targets for many applications of gene delivery, owing to their ability to differentiate into all lineages of the hematopoietic system [4]. HSCs can theoretically maintain the supply of gene-transduced hematopoietic cells throughout a patient's lifespan. Efficient gene delivery into target cells and the maintenance of the biological functions of these cells are therefore crucial to the success of gene therapy.

Various sources of HSCs have been used in research and clinical studies including cord blood (CB), adult bone marrow (BM) and mobilized peripheral blood (PB) HSCs. Although CB HSCs are relatively easier to manipulate *in vitro*, harbor a higher proliferative capacity and are more susceptible to gene transduction [5], [6], adult HSCs are more therapeutically relevant for human gene therapy applications. In particular, granulocyte colony-stimulating factor (G-CSF) mobilized PB stem cells have become the major source of HSC transplantation [7].

Prolonged *ex vivo* manipulation induces entry into the cell cycle resulting in proliferation. This has, in many cases, been shown to decrease the frequency of primitive HSCs in culture and compromise their long-term repopulating ability [8], [9]. Cytokine stimulation of quiescent stem cells prior to transduction aims to induce cell cycling and is an essential requirement for oncoretroviral integration [10], [11]. Although preclinical studies in murine models have shown pre-stimulation can improve the transduction efficiency of reconstituting stem cells, such manipulation can also induce differentiation of HSCs and thereby lead to the loss of their reconstitution ability [8], [12]. Lentiviruses have been shown to transduce both proliferating and non-proliferating cells and are therefore capable of infecting certain types of quiescent cells [13]. This provides the potential to reduce *ex vivo* manipulation of the target cells with the aim of preserving their cellular functions. Although efficient lentiviral transduction of human HSCs may not necessarily require proliferation itself, cytokine stimulation to activate the cells to enter cell cycle may improve their susceptibility to transduction [14], [15]. Improved safety features, such as self inactivating long terminal repeats (SIN-LTRs) and minimal split packaging designs, has led the SIN-LV to become the clinical vector of choice by many researchers. This growing confidence is reflected by the approval for trials using SIN-LV by both the European and American regulatory authorities [16], [17].

Reagents of animal origin, in particular fetal bovine serum (FBS), are commonly used in primary cell culture for both research and clinical trials, particularly in retroviral gene therapy applications [1], [2], [18]. However, there are a number of potential issues regarding safety and efficacy with the use of these reagents. Serum containing medium has been shown to induce/accelerate differentiation of HSCs thereby reducing their multi-potency. Reagents of animal origin also have the potential to induce immunological reactions, may contain adventitious agents and/or harbor the potential risk of prion disease [19]–[21]. For these reasons, it is preferable that *ex vivo* manipulation of human HSCs for clinical application be carried out in the absence of animal products.

Currently, there is great variation among research and clinical groups regarding optimal transduction conditions for efficient lentiviral gene delivery into target HSCs [6], [22]–[25]. In this study, we aim to systematically investigate factors involved in the *ex vivo* transduction of CD34^+^ (G-CSF) mobilized PB HSCs and contribute to the development of a clinically relevant transduction protocol.

## Materials and Methods

### Cells and Cell Culture

Human primary G-CSF mobilized peripheral blood CD34^+^ cells (cryopreserved) were obtained from either Lonza (Basel, Switzerland) or StemCell Technologies (Vancouver, BC, Canada). Briefly, mononuclear cells were obtained from a peripheral blood collected through apheresis. CD34+ cells were then isolated from the MNC population by positive selection using immunomagnetic cell separation procedures. Cells were cryopreserved in a 1.8 ml solution of 50% IMDM, 40% FBS, 10% DMSO (0.2 µm filtered), and shipped on dry ice. All recombinant human cytokines used in primary cell culture, including stem cell factor (SCF), thrombopoietin (TPO), flt3/flk2 ligand (Flt3L) and interleukin-3 (IL-3) were obtained from R&D Systems (Minneapolis, MN, USA). Unless otherwise stated, a standard cytokine mix of SCF, Flt3L and TPO at 50 ng/ml each was used. Various culture media were used, including IMDM (Gibco/Invitrogen, Carlsbad, CA, USA), X-vivo 10 (Lonza, Basel, Switzerland), Stemline (Sigma, St. Louis, MO, USA), Stemline II (Sigma, St. Louis, MO, USA), StemSpan (StemCell Technologies, Vancouver, BC, Canada), HPMG (Cambrex/Lonza, Basel, Switzerland), and CellGro-SCGM (CellGenix, Freiburg, Germany).

### Lentivirus Production and Titre

**Figure 1:**
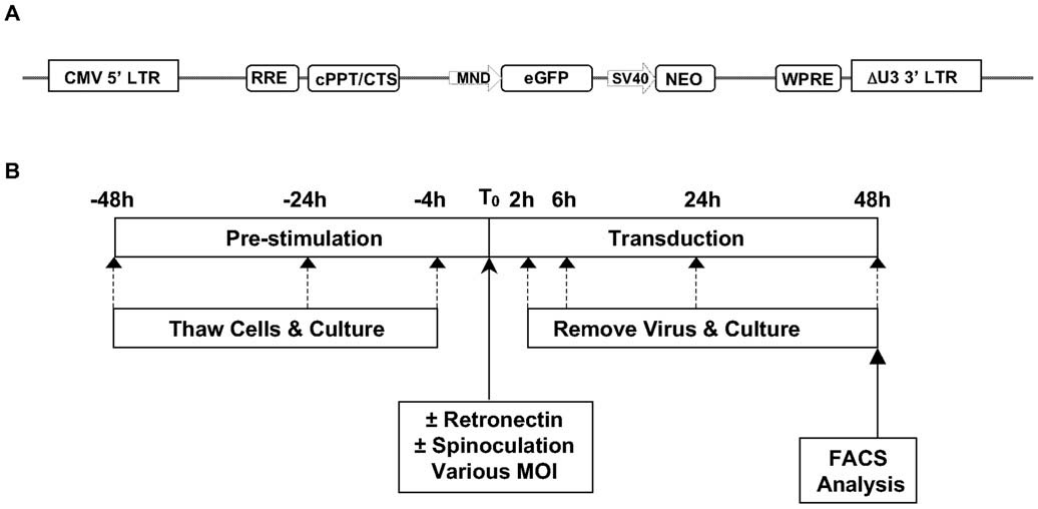
Lentiviral vector and transduction procedures. (A) Schematic diagram of Cell Genesys SIN lentiviral transfer vector pKC.MND.eGFP.neo. CMV 5′ LTR: modified cytomegalovirus 5′ long terminal repeat; RRE: rev response element; cPPT/CTS: central polypurine tract/central termination sequence; MND: myeloid proliferative sarcoma virus promoter; eGFP: enhanced green fluorescent protein; SV40: simian virus 40 promoter; neo: neomycin resistance gene; WPRE: woodchuck post translational regulatory element; ΔU3 LTR: modified U3 long terminal repeat. (B) Schematic diagram of experimental procedures for CD34^+^ transduction. Cells were pre-stimulated for 48, 24 or 4 h prior to transduction at T_0_ at various MOIs, with and without retronectin and/or spinoculation. Virus was washed from the cells 2, 6, 24 or 48 h post transduction, placed back into culture and analysed by flow cytometry 48 h post transduction.

A four-plasmid self-inactivating lentiviral vector system was obtained from Cell Genesys (San Francisco, CA, USA). A transfer vector encoding enhanced green fluorescent protein (pKC.MND-eGFP-neo) was used throughout this study (Figure 1A). GFP, which can be qualitatively and quantitatively measured by flow cytometry, was used as a surrogate in the study as an objective reflection of cells containing the transgene. GFP has estimated half-life of >24 hours [26], exhibiting a high resistance to heat, pH, detergents, organic solvents, and most common proteases [27]–[30]. The stability and ease of detection makes GFP an efficient tool to measure gene marking. Lentivirus was produced in 293AAV cells (Cell Genesys) via calcium phosphate transfection of the plasmids pKC.MND-eGFP-neo; pKgagpol (Gag-Pol); pKrev (Rev) and pK.G (VSVG envelope) at a ratio of mass of 20∶13∶5∶7 respectively. Briefly, cells were seeded at 37.5×10^6^/T225 flask and cultured overnight. The next day cells were transfected in the presence of 10% FBS with 30 µg of transfer vector plasmid and 37.5 µg of packaging mix (19.5 µg Gag-Pol; 7.5 µg Rev; 10.5 µg VSVG). Media was removed 4 h post transfection, cells rinsed and serum free media added. Virus was harvested 24 h later, centrifuged and filtered through 0.2 µm pore size. Virus was harvested in serum free X-vivo 10 unless otherwise stated. Viral titre was determined by transduction of HT1080 cells with 4-fold serial dilutions of VCM (neat to 1/4096) in a 96-well format in triplicate. 72 h post transduction, cells were detached with trypsin and transferred to a 96-well non-tissue culture treated plate for FACS analysis of GFP expression. Viral titre (ivp/ml) was calculated using the percentage of GFP expressing HT1080 cells using the formula as follow: Titre (ivp/ml) equals to (cell number×percentage of GFP^+^ cells×Dilution factor) divided by VCM volume (ml) [31].

### Primary CD34^+^ Cell Culture, Transduction and FACS Analysis

CD34^+^ cells were thawed, counted, assessed for viability and seeded at 0.5 to 2×10^5^ cells/ml in various culture media (as specified in each experiment). Media was supplemented with a number of cytokine combinations including SCF, Flt3L, TPO and IL-3 at different concentrations (as specified in results). Following cytokine pre-stimulation for 4, 24 or 48 h, cells were transduced with a GFP-encoding lentivirus under a number of different conditions: with or without retronectin (5 µg/cm^2^) (Takara Shuzo, Shiga, Japan), with or without spinoculation (600 g for 1 h, 30°C), at various multiplicity of infection (MOI) and differing transduction durations. Cells were harvested 48 h post transduction for FACS analysis. GFP and cell surface marker expression were determined using a FACSCanto II with FACSDiva software (BD Biosciences, San Jose, CA, USA). Transduction efficiency was estimated as the percentage of GFP positive cells. The panel of surface markers used to assess the differentiation state of CD34^+^ cells in culture included CD34, CD38, CD90, CD117, CD135 (BD Biosciences) and CD133 (Miltenyi Biotec, Bergisch Gladbach, Germany). An Aldefluor kit (StemCell Technologies, Vancouver, BC, Canada) was used to measure aldehyde dehydrogenase activity, another marker for HSCs. CountBright beads (Invitrogen, Carlsbad, CA, USA) were added to each FACS tube to allow the estimation of cell concentration. Unless otherwise stated, each experiment was performed with three biological replicates for each condition and FACS analysis for GFP and CD34 expression only was performed in duplicate for each replicate. A summary of experimental procedures is shown in Figure 1B.

### Clonogenic Assay

The clonogenic activities of CD34^+^ cells were assessed following lentiviral transduction. Cells were plated at 1×10^3^/ml in 1% methylcellulose supplemented with SCF 50 ng/ml, GM-CSF 10 ng/ml, IL-3 10 ng/ml and erythropoietin 3 U/ml (Methocult H4434, StemCell Technologies). Granulocyte-macrophage colony-forming units (CFU-GM), erythroid burst-forming units/cluster-forming units (BFU-E/CFU-E), and granulocyte, erythrocyte, megakaryocyte and macrophage colony-forming units (CFU-GEMM) consisting of more than 40 cells were scored at days 10 to 14.

### Statistical Analysis

Statistical analysis was carried out using GraphPad Prism 5 software. Data sets were analysed for statistical significance by One-way ANOVA followed by post hoc Tukey test or Mann-Whitney two tailed test, unless otherwise stated. Results are presented as mean±SD (standard deviation).

## Results

### Comparison of CD34^+^ Cell Growth, Differentiation and Transduction in Serum-containing versus Serum-free Media

**Figure 2:**
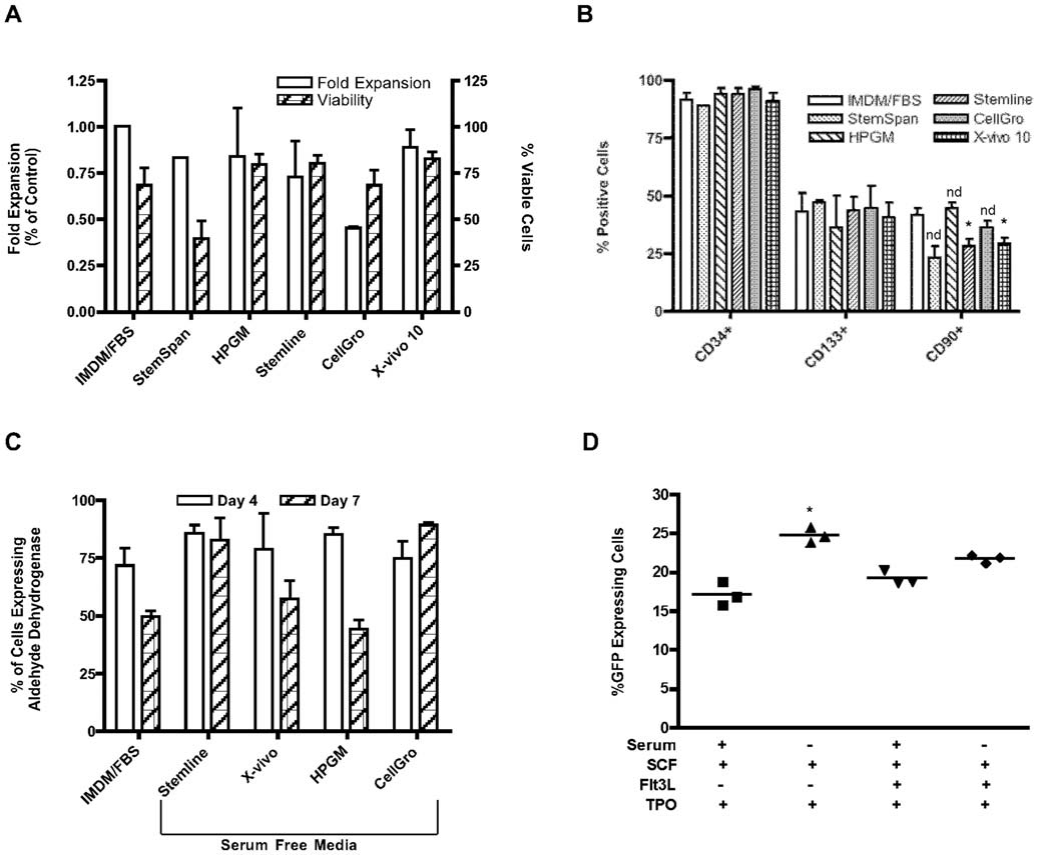
Comparison of CD34^+^ cell growth, differentiation and transduction in serum vs serum-free media. (A) Cell expansion and viability after 4 days in culture in various media. Data collated from independent experiments (n = 3 for IMDM/FBS, Stemline and X-vivo 10; n = 2 for HPGM and CellGro; n = 1 for Stemspan), and normalized to IMDM/FBS control. (B) Expression of primitive markers after 4 days in culture in various media. Data collated from independent experiments (n = 3 for IMDM/FBS, Stemline and X-vivo 10; n = 2 for HPGM and CellGro; n = 1 for Stemspan). (C) Aldehyde dehydrogenase expression expressed as the percentage of aldefluor positive cells on days 4 and 7 of culture in various media. (D) Comparison of transduction efficiency in serum vs serum-free media supplemented with various cytokine combinations. * p<0.05, when comparing serum-free/SCF+TPO to its serum containing counterpart. nd = not determined on data sets where n≤2.

Several serum-free culture media designed for HSCs were tested for their ability to support CD34^+^ growth, maintain multi-potency and to allow efficient gene delivery via lentiviral transduction. Cryopreserved G-CSF mobilized PB CD34^+^ cells were thawed and seeded at 1-2×10^5^cells/ml in various serum free media supplemented with a combination of cytokines, either as recommended by the manufacturer or a “standard” cytokine combination used in our laboratory (SCF, Flt3L and TPO, each at 50 ng/ml). IMDM with 10% FBS, a standard culture medium for primary CD34^+^ cells, was used as the serum-containing control. CD34^+^ cells were assessed via FACS analysis at day 4 and/or day 7 for cell expansion using counting beads and the differentiation status of the cells was determined by the expression of various primitive markers (CD34, CD38, CD133, CD90, CD135, CD117). Aldehyde dehydrogenase, the expression of which is elevated in primitive cells [32]–[34], was also assessed. In general, it was observed that CD38 expression was markedly higher in serum-containing cultures compared to serum-free conditions, possibly due to the presence of retinoids in serum which have been shown to induce CD38 expression [35], [36]. Therefore, the comparison of CD38 expression in serum versus serum-free conditions may not reflect the true maturation state of the cells. The measurement of surface expression of CD135 (Flt3 receptor) and CD117 (c-Kit receptor) was also hampered by internalization of the receptors due to the presence of ligands (Flt3L and SCF) in the culture medium. As such, the expression of CD38, CD135 and CD117 was not analysed in these experiments. Overall, there were no marked differences observed in terms of growth and differentiation between the various culture media assessed. Likewise, the expansion of CD34^+^ cells was not markedly different between the media tested, with the exception of CellGro, which resulted in lower expansion over the 4-day culture period (Figure 2A). Cell viability was also similar across all culture conditions with no significant differences found when compared to the serum containing media, except for Stemspan which yielded a much lower viability of around 40% (Figure 2A). The expression levels of the primitive markers CD34 and CD133 remained similar when cultured in either serum-free or serum-containing media (Figure 2B), although a decrease in CD34 expression was seen for cells cultured in serum containing media after 7 days (72% in the presence of serum versus an average of 98% in serum-free media, data not shown). CD90 expression however, demonstrated a significant decrease when cultured in Stemline and X-Vivo. The prevalence of double positive CD34^bright^/aldefluor^+^ cells was reduced to 15% in serum-containing medium, as compared to 36–62% in serum-free cultures. No marked difference in aldehyde dehydrogenase activity was observed after 4 days in culture (Figure 2C). By day 7 however, the activity decreased to an average of 50% in serum-containing cultures as wells as in two of the serum-free cultures (HGPM and X-vivo 10), but remained high in Stemline (80%) and CellGro (89%). This is not of primary concern as a clinical transduction protocol is unlikely to be longer than 4 days.

The ability of serum free media to support efficient lentiviral transduction was also tested. CD34^+^ cells were pre-stimulated for 48 h in serum free medium (X-vivo 10) or serum containing medium (IMDM+10% FBS) supplemented with either a triple cytokine combination (SCF, TPO and Flt3L all at 50 ng/ml) or a combination of two cytokines as previously used in a phase II retroviral/CD34^+^ clinical trial by this group (SCF 50 ng/ml and TPO at 100 ng/ml) (manuscript submitted). Cells were transduced on retronectin coated plates (5 µg/cm^2^) at an MOI of 9 and transduction efficiency was assessed via GFP expression 48 h post transduction. Overall, a higher level of transduction was seen in serum free cultures: 24.8±1.0% in serum-free versus 17.1±1.5% in serum containing media in the presence of two cytokines (p<0.05); and 22±0.6% in serum-free versus 19.2±0.8% in serum containing media with the triple combination (p>0.05) (Figure 2D). While the mean fluorescence intensity (MFI) of GFP expression was slightly higher in cells cultured in serum containing media (data not shown), the loss of expression of the primitive marker CD34 observed under serum containing conditions with the triple combination was greater (95.8% in serum-free versus 85.6% in the presence of serum, data not shown).

### Comparison of Lentiviral Transduction in Stemline II and X-vivo 10 Serum-free Media

Two serum free media, X-vivo 10 and Stemline II, were chosen for further assessment based on the need for serum-free and animal origin-free reagents, availability of GMP grade products for clinical application and pre-existing data. Stemline II was selected over Stemline due to the manufacturer's claims of a high capacity for the maintenance and expansion of both early (CD34^+^/CD38^−^) and late (CD34^+^/CD38^+^) progenitor cells.

**Figure 3:**
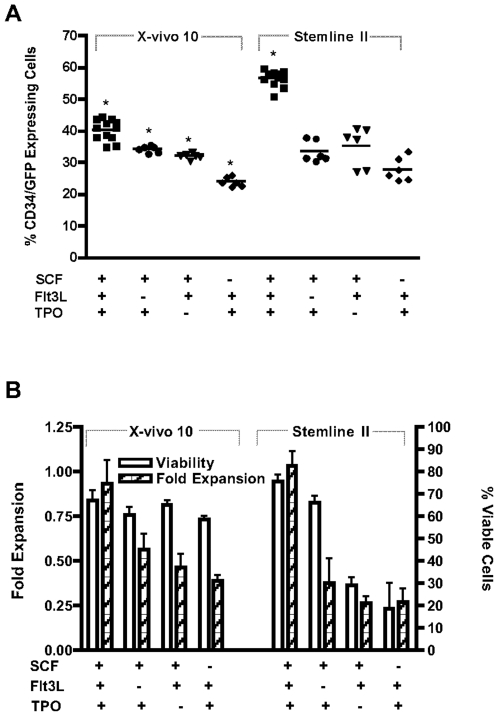
Transduction efficiency of CD34^+^ cells in X-vivo 10 versus Stemline II serum-free media in various cytokine combinations. (A) Transduction efficiency in the CD34^+^ cell population. * p<0.05, when comparing Stemline II/triple cytokines to X-vivo 10/triple cytokines (B) Cell expansion and viability following transduction.

CD34^+^ cells were pre-stimulated for 48 h, in either X-vivo 10 or Stemline II, in the presence of various cytokine combinations (SCF, TPO and Flt3L, all of the three or all combinations of two, each at 50 ng/ml). Cells were then transduced with virus containing media (VCM) containing GFP lentivirus harvested in either X-vivo 10 or Stemline II at an MOI of 9. Transduction was performed on retronectin coated plates via spinoculation. Cells were analyzed by FACS analysis for EGFP expression and cell surface marker expression 48 h post transduction. A higher percentage of transduction was seen in cultures supplemented with the triple cytokine combination as compared to any combination of two (p<0.05), irrespective of the basal media (Figure 3A). This was especially evident in the Stemline II cultures, in which both the transduction efficiency and the MFI of GFP expression was significantly increased with the triple cytokine combination (p<0.05 compared to X-vivo 10) (Figure 3A). Although a higher percentage of transduction was observed in Stemline II cultures, X-vivo 10 appeared to be more robust as less fluctuation in the overall performance of the cells (transduction efficiency and viability) was seen when supported with various cytokine combinations (Figure 3A). The triple cytokine combination also resulted in increased cell numbers (expressed as fold expansion from input cells) in both culture media, while viability remained consistently above 60% across the majority of the culture conditions. The exception was Stemline II supplemented with SCF and Flt3L or Flt3L and TPO combinations, both of which were shown to have viabilities below 30% in one experiment (Figure 3B). No significant difference was observed in the expression of cell surface markers between the various culture conditions with the majority of cells remaining CD34 positive after 96 h in culture (84.4% to 97.4%, data not shown).

X-vivo 10 was chosen for further optimization because of the consistent cell viability and transduction observed with the different media and, unless stated otherwise, was used in all subsequent experiments supplemented with the triple cytokine combination.

### Optimization of Transduction

#### Pre-stimulation

**Figure 4:**
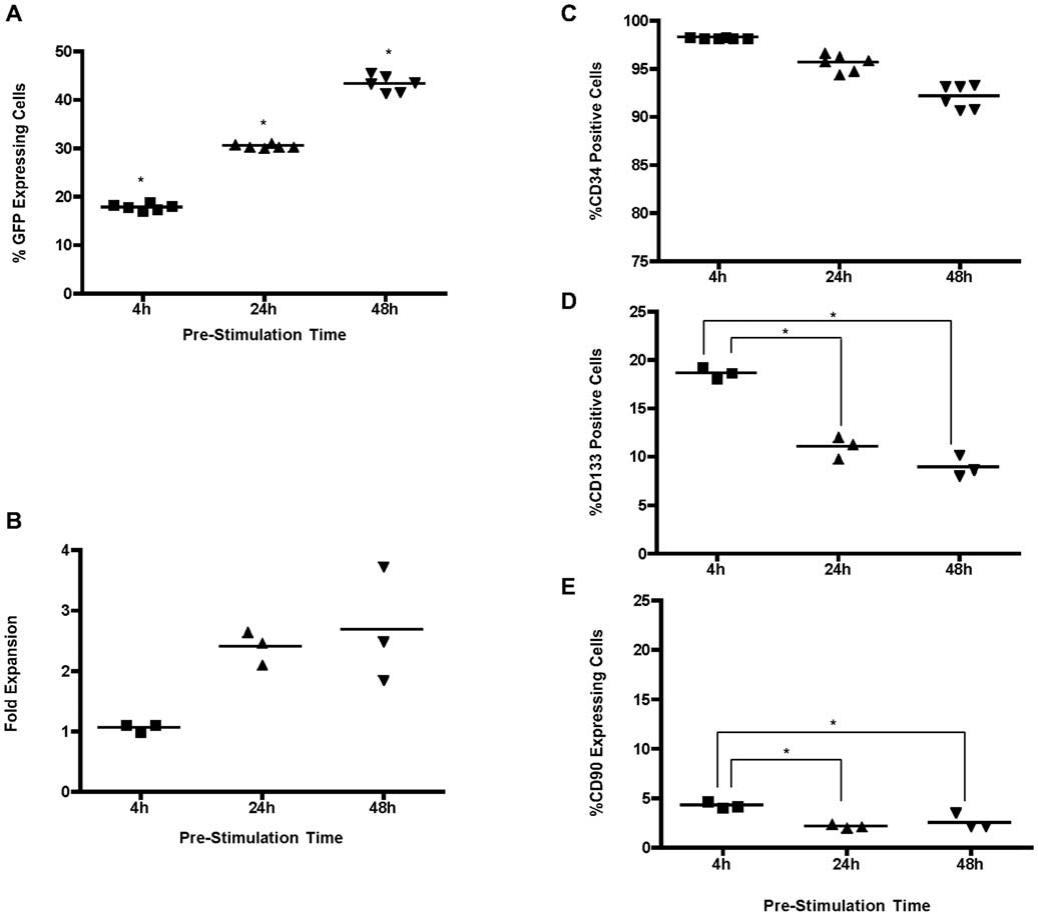
Effects of 4, 24 or 48 h pre-stimulation on CD34^+^ cell transduction, growth and differentiation. (A) Transduction efficiency 48 h post transduction. (B) Expansion of CD34^+^ cells post transduction. (C)–(E) Expression of primitive markers 48 h post transduction, (C) CD34, (D) CD133 and (E) CD90. * p<0.05, when all combinations compared.

Although lentiviruses can transduce both dividing and non-diving cells, several studies have indicated that cytokine pre-stimulation improves the efficiency of lentiviral gene transfer [14], [15]. CD34^+^ cells were pre-stimulated for 4, 24 or 48 h in X-vivo 10 supplemented with the triple cytokine combination. Transduction was carried out on retronectin coated plates using spinoculation at an MOI of 9. Transduction efficiency and the expression of cell surface markers were analyzed 48 h post transduction as in previous experiments. As shown in Figure 4A, transduction efficiency significantly increased (p<0.05 for all combinations) with an increase in pre-stimulation time, both in terms of the percentage of cells transduced and MFI of GFP in the transduced cells. However, the increase in cell expansion in relation to increased pre-stimulation time was not statistically significant (2.7±0.9-fold for 48 h; 2.3±0.3-fold for 24 h; 1.1±0.1-fold for 4 h) (Figure 4B). No differences were observed in the levels of CD34 expression between the pre-stimulation times however, CD133 and CD90 expression was significantly decreased following 24 h and 48 h pre-stimulation (Figure 4C–E). It was determined that 24 h pre-stimulation provided a suitable compromise for pre-stimulation time and transduction efficiency and was used in subsequent experiments.

#### Facilitation of Transduction: Spinoculation and/or Retronectin

**Figure 5:**
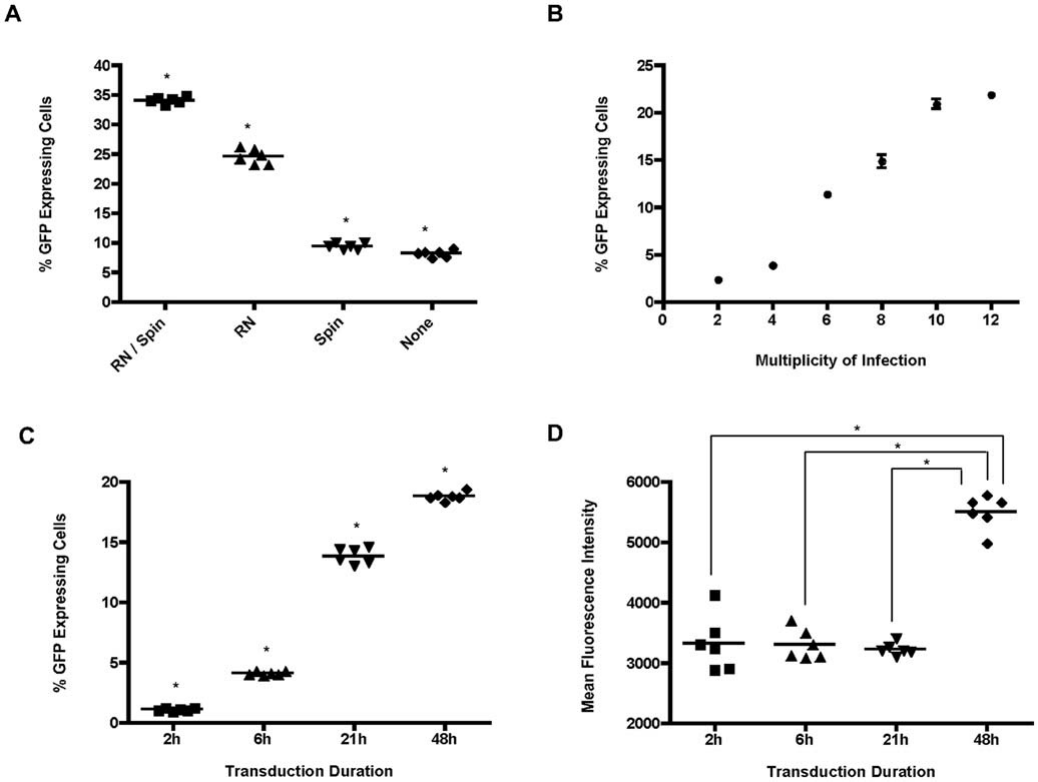
Assessment of CD34^+^ transduction methods and duration. (A) Transduction efficiency using retronectin and/or spinoculation. RN/Spin; retronectin and spinoculation; RN: retronectin only; Spin; spinoculation only; none: no retronecin or spinoculation. * p<0.05, when all combinations compared. (B) Transduction efficiency and GFP expression levels of cells transduced with a range of MOIs (0, 2, 4, 6, 8, 10, 12), R^2^ = 0.9655 (Pearson Correlation). (C) & (D) Effects of transduction duration on efficiency. (C) Transduction efficiency, * p<0.05 for all combinations and (D) GFP expression of transduced cells following various transduction durations (2, 6, 24 and 48 h). * p<0.05, when 2, 6 and 21 h are compared to 48 h.

The necessity for the use of retronectin and spinoculation to facilitate transduction was assessed to further streamline the transduction protocol for possible clinical application. Following 24 h pre-stimulation (X-vivo 10, triple cytokine combination), CD34^+^ cells were transduced on retronectin coated plates (5 µg/cm^2^) or tissue culture treated plates with and without spinoculation. As shown in Figure 5A, significant differences were observed between all methods tested (p<0.05). Retronectin combined with spinoculation resulted in the highest transduction efficiency both in terms of the percentage of cells transduced (34.1±0.5%) and MFI of GFP expression in the transduced cells. Transduction using retronectin alone resulted in 24.6±1.3% transduction. There was no advantage seen with spinoculation alone as the transduction efficiency was comparable to the transduction levels of 10% achieved in the untreated tissue culture plate and spinoculation was not investigated further.

#### Multiplicity of Infection

The effect of increasing the MOI on transduction of CD34^+^ cells was assessed. Cells were pre-stimulated for 24 h and transduced on retronectin coated plates using 2-fold serial dilutions of VCM resulting in MOIs ranging from 2 to 12. When analysed 48 h post transduction, an increase in the percentage of cells transduced correlated well (R^2^ = 0.9655) with the increase in MOI (Figure 5B). This was accompanied by an increase in the MFI of GFP expression (data not shown), suggesting an increase in the amount of GFP produced by the cells, thereby possibly indicating higher copy numbers in those cells transduced with higher MOIs.

#### Transduction Duration

The duration of time for which cells are exposed to virus was also investigated. CD34^+^ cells were transduced on retronectin coated plates using an MOI of 5 following 24 h pre-stimulation. The cells were cultured in VCM for 2, 6, 24 and 48 h, after which time the cells were washed and put back into culture in fresh growth medium. Transdution efficiency was analyzed 48 h post initiation of transduction. The percentage of transduced cells was found to increase significantly with a longer transduction duration, with 18.8±0.4% of cells expressing GFP following 48 h transduction and just over 1% for 2 h transduction (p<0.05, Figure 5C). Despite the increase in the percentage of cells transduced, the MFI remained similar for the 2, 6 and 24 h transductions, with a significant increase seen only for the cells transduced for the 48 h time period (p<0.05 when comparing 48 h time period to 2 h, 6 h and 24 h, Figure 5D). This suggests the possibility of a higher copy number per cell occurring with a transduction period longer than 24 h.

### Comparison of CD34^+^ Cell Growth and Transduction with Various Cytokine Combinations

**Figure 6:**
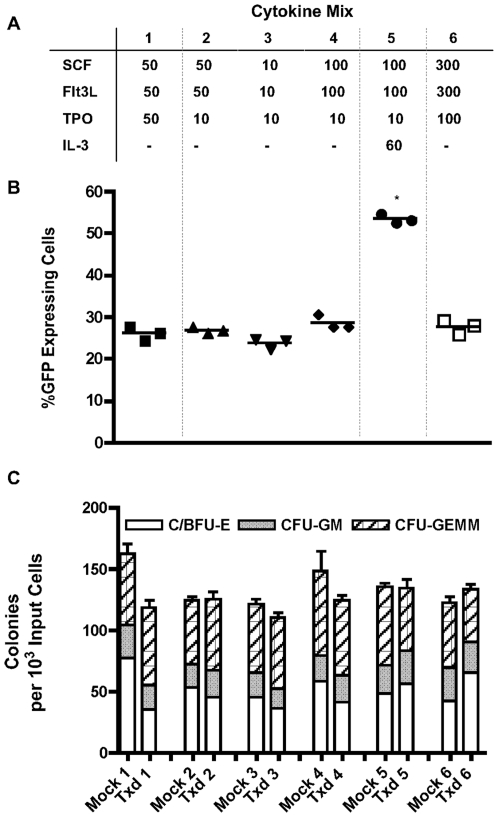
Comparison of CD34^+^ cell growth and transduction with various cytokine combinations. (A) Cytokine combinations used, ng/mL (B) Transduction efficiency in various cytokine combinations. * p<0.05, when compared to all other cytokine combinations (C) Clonogenic potential. Mock: non-transduced cells; Txd: transduced cells; C/BFU-E: Colony/blast forming units – erythroid; CFU-GM: Colony forming unit – granulocyte/macrophage; CFU-GEMM: Colony forming unit – granuloycte, erythroid, macrophage, megakaryocyte.

Six different combinations of cytokines previously reported [1]–[3], [24], [25] were tested as shown in Figure 6A. CD34^+^ cells were cultured and transduced in these cytokine combinations under standard conditions (24 h prestimulation, 48 h transduction on retronectin coated plates) at an MOI of 9 and analysed for GFP and surface marker expression 48 h post transduction. Transduced cells were then plated into methylcellulose cultures to measure the effects of each cytokine combination on the clonogenic capacity of the cells. Similar levels of transduction were seen with all combinations (ranging from 23.7% to 28.6%) with the exception of the IL-3 containing cytokine mix, in which transduction was significantly higher at 53.4±1.2% (Figure 6B). This increase in the transduction level in the IL-3 containing culture was accompanied by a slight decrease in CD34 expression along with an increased total cell number as compared to all other cytokine combinations (0.56×10^6^ for the IL-3 containing culture compared to 0.20–0.25×10^6^ for all other conditions, data not shown). No marked difference in cell viability was observed between the conditions. Although the addition of IL-3 increased the transduction level, it may negatively act to induce cell differentiation *ex vivo*, thereby reducing the multi-potency of the CD34^+^ cells. Interestingly, increased cytokine concentrations (up to 300 ng/ml for SCF and Flt3L; 100 ng/ml for TPO) did not provide any advantage over the low concentration conditions (10 ng/ml SCF, Flt3L and TPO) in terms of either transduction (Figure 6B) or clonogenic activity of the cells, determined as total colony number and ratio of different types of colonies (Figure 6C).

## Discussion

Gene therapy is a relatively new therapeutic approach with early stage clinical development still under investigation. As such, no standard procedures exist regarding target cell *ex vivo* manipulation. Research and/or clinical applications have used a wide variety of culture components and protocols. Safety and efficacy are of primary concern with such cell therapies and some current trials are incorporating the use of serum-free culture conditions for the *ex vivo* manipulation of HSCs. This study provides a systematic investigation into parameters involved in *ex vivo* culture and transduction of CD34^+^ HSCs for the development of a clinically relevant protocol.

Vector design is continually being improved to address serious safety concerns such as insertional oncogenesis and the generation of replication competent virus. Murine leukemia virus (MLV)–based gammaretroviral vectors and human immunodeficiency virus (HIV)–based lentiviral vectors are the two most commonly used retroviral vector systems used in gene therapy. Theoretically, both of these integrative viral vectors may trigger oncogenesis as a consequence of insertional mutagenesis, as shown in early clinical trials in which gamma-retrovirus caused overt leukemia in some patients [37]–[39]. More recent integration studies from various groups have demonstrated a clear difference in integration patterns between these two vector systems [40]–[43]. While gamma-retrovirus demonstrates a bias towards integrating near transcription start sites and certain cell-cycle genes and oncogenes, the HIV-based Lentivirus tends to integrate preferentially within highly expressed genes [40]–[44]. For these reasons it is speculated that the retroviral system may be more prone to adverse gene activation, and such gene activation results from the activation of the downstream genes by the long terminal repeats (LTRs) within these vectors [45], [46]. A recent study by Montini *et al*. identified the transcriptionally active LTR as the major determinant of genotoxicity and validated the improved safety conferred by SIN LTR design [47]. For these reasons, vectors now incorporate self-inactivating LTRs (SIN-LTRs), thereby reducing the potential for promoter activation [48]. Another great safety concern for clinical gene therapy application is the potential for the production of replication competent retrovirus/lentivirus (RCR/RCL). As the production of RCR/RCL is believed to occur through homologous recombination between overlapping sequences, the functional components of the viral genomes are divided onto separate expression plasmids to minimize this risk [49], [50]. These new split-function packaging designs are inherently safer as multiple, ordered recombination events would be required to generate a replication competent virus. These improved safety features often make lentivirus the preferred vehicle for clinical gene therapy by many researchers and may become the new standard acceptable by regulatory authorities in the near future. Currently, several clinical trials employing lentiviral vectors, including a triple therapy for the treatment of HIV have been approved by FDA (collaborative study between Benitec and the City of Hope, 16), and an adrenoleukodystrophy (ALD) trial by EMEA (European Medicinal Agency, 17).

We demonstrated that a SIN lentiviral vector can readily transduce G-CSF mobilized PB CD34^+^ cells with minimal *ex vivo* manipulation under serum-free culture conditions, without altering their proliferation potential or ability to maintain multi-lineage differentiation. The removal of FBS from cell culture eliminates the safety concerns associated with the use of such products and provides for a more consistent outcome by removing likely batch variations in FBS. Serum-free culture conditions have been increasingly used for clinical gene transfer [3], [54] and our results demonstrate that serum-free culture does not impair CD34^+^ cell survival or growth *in vitro* and maintains the primitiveness of these cells as measured by aldehyde dehydrogenase expression. This study also demonstrates that serum-free culture supports lentiviral gene delivery into primary cells as well as, if not better, than serum containing media. This is further evidence to support the use of serum-free *ex vivo* culture as a practical approach for gene therapy applications.

To maintain the biological function of HSCs, namely self-renewal and multipotency, minimizing *ex vivo* manipulation by reducing culture time and the concentration of stimulatory cytokines in the culture media is an important step. Minimal cytokine stimulation of HSCs may help preserve stem cell function and provide for the effective “activation” of stem cells that renders them susceptible to transduction. Although other reports demonstrate transduction of human CD34^+^ cells in the absence of cytokine pre-stimulation, increased levels of gene transfer were consistently observed if the cells were cultured with well-defined cytokines [55], [56]. A recent study has shown that proteasome activity, which limits the efficiency of HSC transduction by lentiviral vectors, is down-regulated in hematopoietic progenitors by cytokine prestimulation [57]. In our study, although higher transduction was achieved following 48 h pre-stimulation, the corresponding decrease in the surface expression of CD90 and CD133 indicates a loss of primitivity. Four hour pre-stimulation on the other hand, demonstrated substantially reduced transduction, more than two fold lower than 48 h pre-stimulated cells, yet surface expression of primitive markers remained relatively high. Interestingly, our data also showed that higher cytokine concentrations do not provide for improved transduction efficiency over that achieved with just 10 ng/ml of SCF, Flt3L and TPO. It must be considered that the experiments in this report were performed with cryopreserved and not fresh HSCs which would be the likely target cells for gene delivery in clinical setting. It remains possible that efficient transduction may be achieved with short (4 h) to no pre-stimulation in a low concentration (10 ng/ml) of cytokines in freshly isolated HSCs, and this warrants further testing in clinical studies. While an assessment of the bone marrow repopulation capacity of transduced cells would be invaluable in evaluation of the preservation of biological function of HSCs, the inherent lack T cell development in the standard NOD/SCID mouse model of human hematopoietic reconstitution, may have limited biological relevance for therapies targeting the T-cell Lineage.

It has been demonstrated that the MOI at the time of transduction closely correlates with the copy number in target cells [58], [59]. Transduction of HSCs using a low MOI to achieve a low copy number per cell is preferable for clinical trial protocols to minimize the risk of insertional mutagenesis. In the present study, we demonstrated that transduction correlated with MOI, both in terms of the percentage of cells transduced and the MFI of GFP expression in transduced cells. This supports previous studies to indicate that an increased MOI may increase the total number of transduced cells but also increase the number of integrated copies per cell. Other studies have shown that lentiviral vectors transiently arrest transduced cells when used at high concentrations and resulted in a marked decrease in the growth rate of the cells [60], [61]. The lack of observed differences in cell growth and viability in our study may be explained by the relatively low range of MOI (between 2 to 12, due to the limitations of cell number and viral titre) used throughout the study, which is markedly lower than those reported by some other groups [24], [25], [62]. Another important factor to be taken into consideration is the duration in which the cells are in contact with the virus. The time frame should be long enough to enable infection of a maximum number of cells, however, prolonged exposure may cause unwanted cellular toxicity and may also increase the chance of multiple integrations [63], [64]. From our observations, the percentage of transduced cells increased with an increase in exposure time, but the MFI of GFP expression in the transduced cells remained similar for exposures of up to 24 h. However, transduction times as long as 48 h not only resulted in an increased percentage of marked cells, but also exhibited an increase in MFI, indicating a possible increase in target cell copy number. The use of low MOIs combined with a shorter virus exposure times of up to 24 h may therefore be preferable for transduction conditions for clinical protocols.

In summary, our study investigated the possibility of minimizing the *ex vivo* manipulation of primary CD34^+^ cells for use in clinical applications with the primary aim of maintaining safety and efficacy. Critical parameters, such as culture conditions free of serum and other products of animal origin, pre-stimulation and cytokine combinations, transduction, MOI and virus exposure period were assessed. Our study supports the practicality of using a transduction protocol that employs short to no pre-stimulation with low cytokine concentrations, low MOI and short virus exposure times under serum-free culture conditions. Further detailed analysis of integrated viral copy number and evaluation of transduction protocols would be required in the context of the intended clinical application along with functionality testing for the transduced cells.
